# Integrated multi‐regional multiomic profiling of breast phyllodes tumours reveals peritumoural immune activation and stromal remodelling

**DOI:** 10.1002/ctm2.70644

**Published:** 2026-03-31

**Authors:** Tian‐Qi Gu, Lei Wang, Xiang‐Rong Wu, Qiang Zheng, Fei‐Lin Qu, Chao Chen, Gen‐Hong Di, Zhi‐Ming Shao, A‐Yong Cao

**Affiliations:** ^1^ Key Laboratory of Breast Cancer in Shanghai Department of Breast Surgery Fudan University Shanghai Cancer Center Shanghai China; ^2^ Department of Oncology Shanghai Medical College Fudan University Shanghai China; ^3^ Department of Pathology Fudan University Shanghai Cancer Center Shanghai China

**Keywords:** angiogenesis, breast phyllodes tumour, extracellular matrix remodelling, immune exclusion, peritumoural microenvironment

## Abstract

**Background:**

Breast malignant phyllodes tumours are rare fibroepithelial neoplasms arising from periductal stromal cells, characterized by rapid progression and a high recurrence rate. The poor prognosis largely stems from the lack of effective therapeutic strategies, underscoring the insufficient understanding of their molecular mechanisms and therapeutic targets. Moreover, most previous studies have mainly focused on the tumour core, while the molecular features of the surrounding peritumoural tissue remain insufficiently explored.

**Methods:**

To address this problem, we collected 66 phyllodes tumour specimens from 22 patients, including benign, borderline and malignant subtypes. For each case, paired samples were obtained from the tumour core and peritumoural regions. Multi‐regional genomic, transcriptomic and digital pathology analyses were performed to characterize spatial patterns of tumour evolution. In addition, multiplex immunofluorescence was applied to validate the spatial distribution of key cellular and molecular features.

**Results:**

Malignant phyllodes tumours exhibited markedly enhanced proliferative activity compared with benign and borderline counterparts. Malignant tumours were also characterized by a distinctly immune activated peritumoural niche encasing an immune excluded tumour core. Specifically, the peritumoural regions displayed abundant lymphocyte infiltration and close immune cell clustering, whereas the intratumoral compartments were largely devoid of immune cells. Enhanced angiogenesis and collagen remodelling were observed in the peritumoural compartment. In malignant phyllodes tumour patients, a more pronounced spatial immune segregation phenotype may be associated with a lower risk of recurrence.

**Conclusion:**

These results provide an integrated view of phyllodes tumour progression and identify immune exclusion as a defining feature of malignancy. The unique biological characteristics of the peritumoural region may serve as valuable therapeutic targets, offering potential for combined anti‐angiogenesis agents and immunotherapy strategies to overcome the immune excluded microenvironment of malignant phyllodes tumours.

## Graphical abstract

- Malignant phyllodes tumours show markedly higher proliferative activity than benign and borderline tumours.
- Malignant phyllodes display an immune activated peritumoural region but an immune excluded tumour core.
- In malignant cases, a more pronounced immune segregation phenotype may be associated with a lower risk of recurrence.

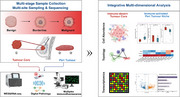

## 1 INTRODUCTION

Breast phyllodes tumours are rare biphasic fibroepithelial neoplasms accounting for < 1% of all breast tumours, characterized by the proliferation of stromal components.^1^ Histologically, they are classified into benign, borderline and malignant subtypes according to stromal cellularity, atypia, mitotic activity and tumour margins.^2^ Although most breast phyllodes tumours are managed successfully with local excision, malignant phyllodes tumours exhibit rapid growth, frequent local recurrence and potential for distant metastasis, posing significant clinical challenges.^3^, ^4^ For instance, malignant phyllodes tumours exhibit a high local recurrence rate of 14%–21%.^5^ Patients who experience recurrence are largely refractory to conventional radiotherapy and chemotherapy.^6^, ^7^ This phenomenon may be attributed to the limited understanding of the molecular landscape of malignant phyllodes tumours, which has hampered the development of precision therapies. Thus, comprehensive molecular investigations are urgently needed to uncover actionable targets and improve clinical outcomes.

Deciphering the molecular and pathological alterations that occur during the progression from benign through borderline to malignant phyllodes tumours is critical for uncovering the molecular feature of this tumour entity. Previous investigations have explored the malignant progression of breast phyllodes tumours from multiple perspectives, including genomic evolution,^8^ stromal cells alteration^9^ and microenvironmental cells reprogramming.^10^ However, most of these studies have focused primarily on the tumour mass itself, particularly the so called “tumour core”. However, the biological significance of the surrounding peritumoural tissue has received comparatively little attention.

The peritumoural region, defined as the microenvironment adjacent to the tumour boundary, appears histologically normal but often displays unique molecular and phenotypic alterations.^11^, ^12^ These regions have been shown to display changes in stromal activation, gene expression, and epigenetic states, reflecting a “field cancerization” effect in which tumour induced molecular perturbations extend beyond the visible tumour margins.^13^ Increasing evidence highlights the clinical relevance of peritumoural tissues: immune profiles of peritumoural versus intratumoral regions better predict prognosis in early stage non‐small cell lung cancer.^14^ Additionally, transcriptomic signatures from peritumoural regions of colorectal tumours correlate more strongly with responses to immunotherapy than those within tumour cores.^15^ Despite these findings across multiple tumour types, no study to date has systematically characterized the peritumoural landscape of breast phyllodes tumours, leaving an important gap in our understanding of how tumour‐stromal interactions shape malignant evolution.

In this study, we performed a comprehensive multi‐regional analysis of breast phyllodes tumours to uncover the molecular and spatial determinants of malignant progression. By integrating transcriptomic profiling, digital pathology, and spatial computational analyses of paired tumour core and peritumoural tissues across benign, borderline, and malignant stages, we systematically delineated the evolution of tumour biology and its surrounding microenvironment.

## 2 RESULTS

### 2.1 Multi‐dimensional and Multi‐omics Investigation of Breast Phyllodes Tumours

**FIGURE 1:**
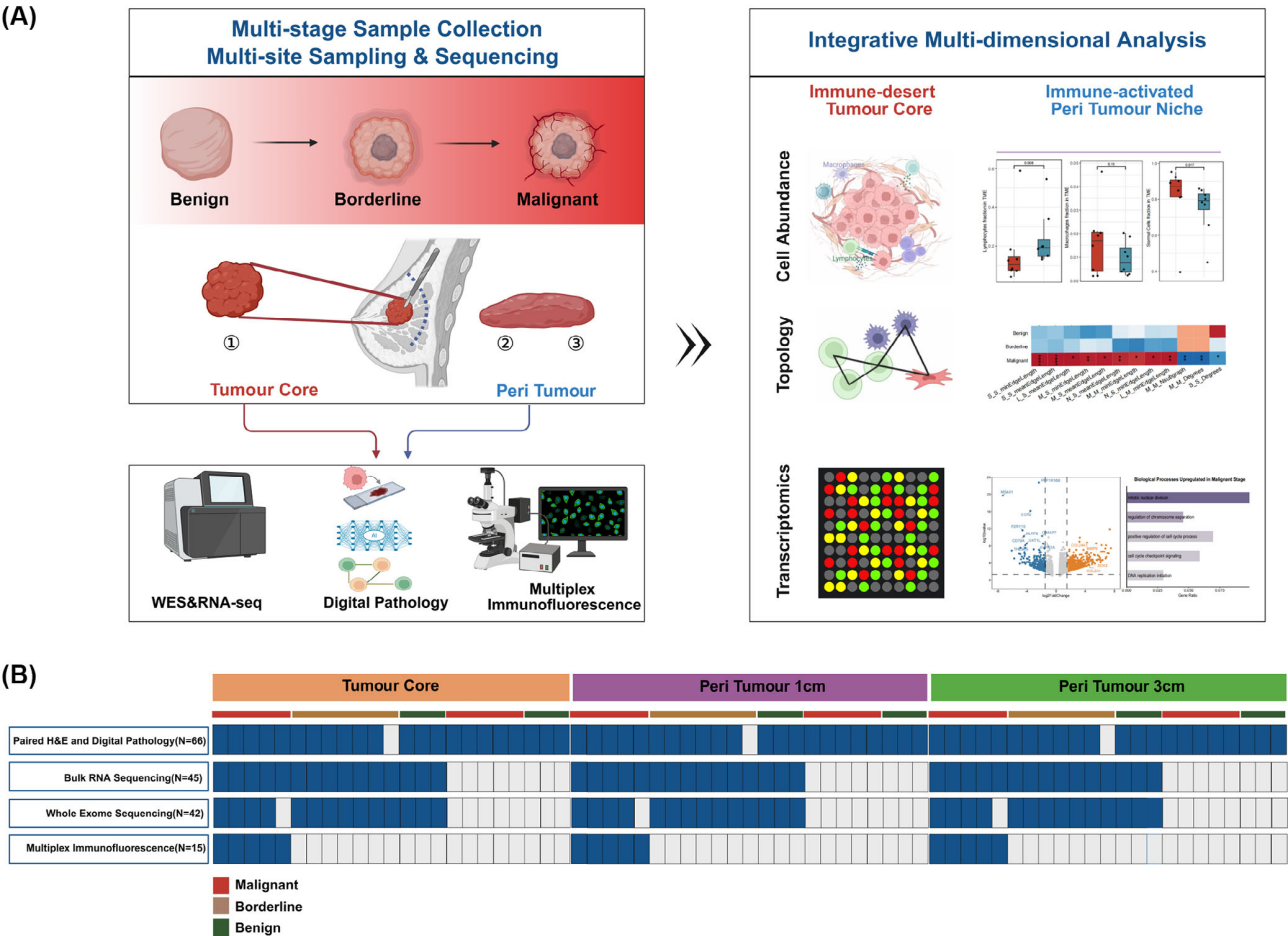
Study design and cohort composition. (A) Schematic overview of the study workflow. (B) Heatmap summarizing the composition of cohorts included in this study.

To comprehensively explore the biological characteristics and pathological features of breast phyllodes tumours from both tumour and peritumoural perspectives, we retrospectively collected 66 samples from 22 patients diagnosed with benign, borderline, or malignant phyllodes tumours. For each patient, tissue specimens were obtained from three sites: the tumour core, tissue located 1 cm from the tumour margin, and tissue located 3 cm from the tumour margin. The 3 cm site was selected to represent relatively normal peritumoural tissue, as previous studies have recommended a surgical margin of ≥1 cm for complete excision of phyllodes tumours.^16^ Two independent pathologists evaluated the corresponding H&E sections and confirmed that the peritumoural specimens used for downstream analyses were free of microscopic tumour infiltration on morphology, thereby ensuring that regions sampled beyond this distance are unlikely to contain residual tumour cells. Among these, 45 samples from 15 patients were subjected to RNA sequencing (RNA‐seq) and whole exome sequencing (WES). In addition, histopathological slides from these patients were systematically reviewed and digitized for subsequent computational pathology analyses (Figure 1A). Collectively, this dataset provides a comprehensive multi‐phase, multi‐site, and multi‐omics resource, laying the foundation for subsequent in‐depth analyses of breast phyllodes tumours (Figure 1B).

### 2.2 Distinct Molecular and Spatial Features of the Core Region in Malignant Phyllodes Tumours

**FIGURE 2:**
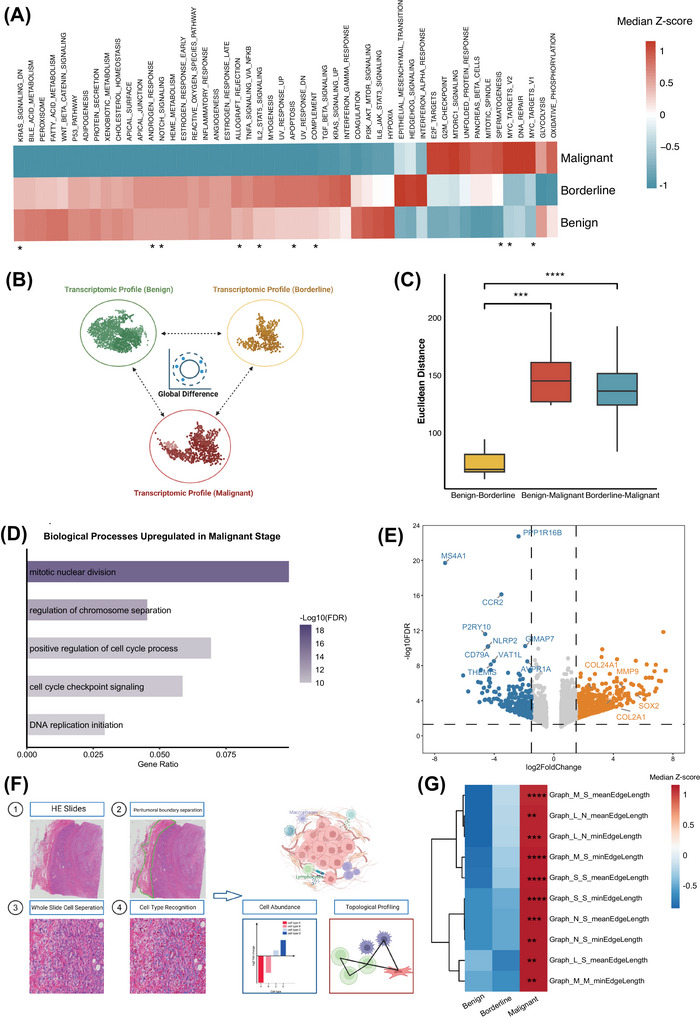
Landscape of tumour core tissues in breast phyllodes tumours. (A) Hallmark pathway enrichment analysis comparing transcriptomic profiles among benign, borderline, and malignant phyllodes tumours. (B) Schematic illustration of global transcriptomic distance calculation across tumour stages. (C) Quantitative comparison of global transcriptomic distances among the three stages. (D) Gene Ontology (GO) enrichment analysis highlighting biological pathways upregulated in malignant tumour cores. (E) Volcano plot depicting upregulated and downregulated genes in malignant versus borderline tumour cores. Coloured points indicate genes with significant changes (false discovery rate < 0.05, |log2FoldChange| > 1.5). (F) Workflow schematic showing digital pathology and deep learning‐based identification of cell abundance and spatial topological interactions. (G) Heatmap displaying spatial topological features across different tumour stages. Statistical significance is denoted as *p* < 0.05 (*), *p* < 0.01 (**), *p* < 0.001 (***), *p *< 0.0001 (****); ns, not significant.

Given that the tumour core represents the primary site of neoplastic proliferation and dictates the overall malignant potential, we first investigated the molecular alterations that occur across different histological stages of phyllodes tumours, following analytic strategies used in previous studies.^17^, ^18^ To gain a global understanding of the biological behaviours associated with each stage, we applied the single sample gene set enrichment analysis (ssGSEA) algorithm to evaluate the enrichment of 50 hallmark oncogenic signalling pathways in tumour tissues of varying grades (Figure 2A). In general, benign and borderline phyllodes tumours displayed similar biological profiles, whereas malignant phyllodes tumours showed marked enrichment of proliferation related pathways, consistent with their aggressive phenotype. To further assess global transcriptional divergence among different stages, we calculated the pairwise Euclidean distances between the corresponding transcriptomes^19^ (Figure 2B). Consistent with the Hallmark pathway analysis, malignant phyllodes tumours exhibited substantial global transcriptomic differences from both benign and borderline tumours, while the discrepancy between benign and borderline tumours remained relatively small (Figure 2C).

At the pathway level, Gene Ontology (GO) enrichment analysis revealed prominent activation of DNA replication and cell cycle pathways in malignant phyllodes tumours (Figure 2D). In contrast, angiogenesis related pathways were specifically enriched in borderline tumours compared with benign ones, suggesting that the borderline stage represents a transitional state characterized by emerging invasive potential^20^ (Figure S1A,B). Consistent with these pathway level findings, at the molecular level, malignant phyllodes tumours showed significant upregulation of stemness associated genes, including *SOX2*. Notably, genes involved in collagen organization and fibrosis, such as *COL2A1* and *COL24A1*, were also markedly upregulated (Figure 2E), indicating a strong association between malignant progression and extracellular matrix remodelling.

WES further revealed that *MED12* mutations were the most frequent genetic alterations in phyllodes tumours, in agreement with previous reports^8^ (Figure S1C). We stratified patients by *MED12*‐mutant versus *MED12*‐wildtype status and evaluated transcriptomic differences. No global transcriptomic separation between *MED12*‐mutant and *MED12*‐wildtype cases was observed when analysing all phyllodes tumours across stages together. However, when restricting the analysis to malignant phyllodes tumours, we found that *MED12*‐mutant tumours exhibited significant upregulation of interferon (IFN) related signalling genes compared with *MED12*‐wildtype tumours. This observation is consistent with prior reports suggesting a close link between *MED12* alterations and immune related transcriptional programs^21^, ^22^ (Figure S1D). However, due to limited sample size, no statistically significant differences in mutation frequency were observed across the three pathological stages. Prior literature suggests that genetic alterations in adjacent tissue may be biologically informative.^23^ We further examined whether *MED12* mutations show spatial heterogeneity. We observed that the mutation frequency of *MED12* was lower in peritumoural samples than in tumour core samples, supporting the interpretation that *MED12* alterations are more tumour enriched. In contrast, mutations in mucin related genes (e.g., *MUC16*) were enriched in peritumoural tissue, suggesting that the genomic landscape of the peritumoural region may reflect distinct selective pressures or tissue specific processes (Figure S1E). Copy number amplification patterns across tumour stages were analysed. Malignant phyllodes tumours harboured a markedly higher burden of copy number amplifications compared with benign and borderline tumours, suggesting that increased CNA burden may contribute to the high proliferative capacity and aggressive biology of malignant phyllodes tumours (Figure S1F,G).

Advances in digital pathology and deep learning‐based image analysis have enabled spatial profiling of tumour architecture. Using high resolution digital slides, we applied a self‐developed artificial intelligence pipeline to identify key microenvironmental cell types (i.e., lymphocytes, macrophages, neutrophils and stromal cells) and to quantify their topological interactions^24^, ^25^ (Figure 2F; also see Methods). Cell‐type assignments made by two independent pathologists further confirmed the reliability of this algorithm (Figure S2A,B). The overall abundance of microenvironmental cell types did not significantly differ among the three stages (Figure S2C–F). Interestingly, topological interaction patterns demonstrated that core region in malignant tumours displayed increased intercellular distance, potentially reflecting an immune excluded and loosely organized stromal landscape (Figure 2G). Collectively, these findings reveal that malignant phyllodes tumours are characterized by distinctive molecular hallmarks and a unique spatial topology within the tumour microenvironment.

### 2.3 Characteristics of the Peritumoural Region in Breast Phyllodes Tumours

**FIGURE 3:**
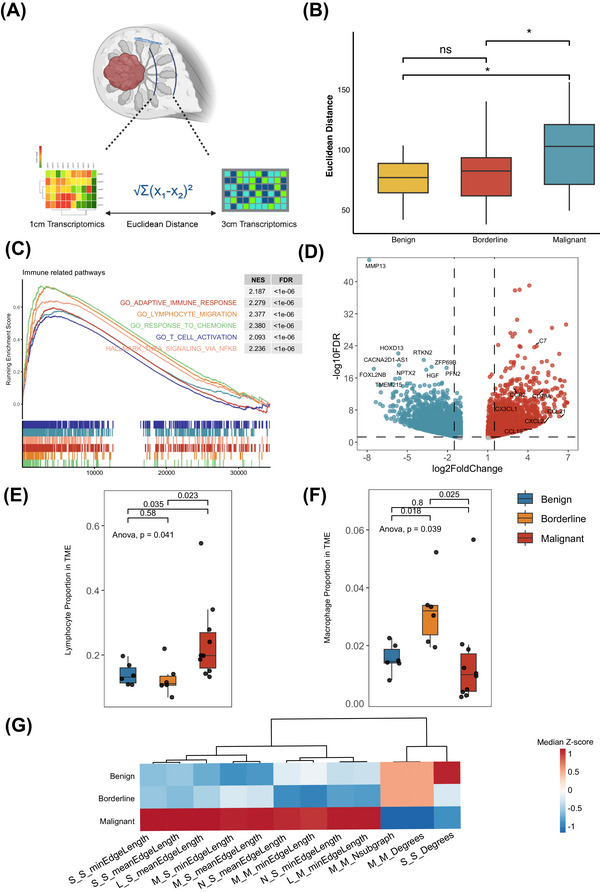
Landscape of peritumoural tissues in breast phyllodes tumours. (A) Schematic illustration of Euclidean distance calculation between transcriptomes derived from peritumoural tissues located 1 and 3 cm from the tumour margin in benign, borderline, and malignant phyllodes tumours. (B) Comparison of Euclidean distances between 1 and 3 cm peritumoural regions among the three histological stages. (C) Gene set enrichment analysis (GSEA) identifying immune related pathways upregulated in malignant peritumoural tissues. (D) Volcano plot showing upregulated and downregulated genes in malignant peritumoural tissues compared with peritumoural tissues from benign and borderline tumours. (E) Quantification of lymphocyte abundance across peritumoural tissues of different tumour stages. (F) Proportion of macrophages in the peritumoural regions among the three stages. (G) Heatmaps displaying the spatial topological features of peritumoural tissues at different stages. Statistical significance is denoted as *p* < 0.05 (*), *p *< 0.01 (**), *p* < 0.001 (***), *p* < 0.0001 (****); ns, not significant.

Besides core region, the peritumoural region plays a pivotal role in tumour progression, invasion, and recurrence, as interactions between tumour cells and the surrounding stroma critically shape the local microenvironment and influence disease behavior.^26^, ^27^ The availability of multi‐site sampling in our study enabled an in‐depth characterization of the peritumoural tissues, which may display biological and molecular properties distinct from those of the tumour core. We first calculated the Euclidean distances between the transcriptomes of the peritumoural regions located 1 and 3 cm from the tumour margin across different histological stages (Figure 3A). Notably, malignant phyllodes tumours exhibited substantially greater transcriptomic divergence between these two peritumoural sites compared with benign and borderline tumours, suggesting a stronger field effect of malignant lesions on the surrounding tissue (Figure 3B). Next, we explored the molecular signatures and pathways upregulated in the peritumoural tissue of malignant tumours. Gene set enrichment analysis (GSEA) revealed significant enrichment of adaptive immune activation, lymphocyte migration, and immune cell signalling pathways in the malignant peritumoural region compared with benign and borderline stages (Figure 3C). Chemokine related genes such as *CXCL2*, *CCL19* and *CCL21* were significantly overexpressed (Figure 3D). These findings indicate that malignant phyllodes tumours induce a distinct immune activated peritumoural microenvironment.

To further integrate the transcriptomic profiles with histopathological features, we delineated the tumour core and peritumoural areas on digital pathology slides and applied our deep learning‐based pipeline for cellular composition and spatial topology analysis. As expected, the peritumoural region of malignant tumours exhibited significantly higher lymphocyte abundance than that of benign or borderline tumours (Figure 3E). Interestingly, we also observed a marked enrichment of macrophages in the peritumoural areas of borderline tumours (Figure 3F), suggesting a stage specific immune landscape. Consistent with our observations in the tumour core, topological analyses showed that cellular distributions in malignant peritumoural tissues were spatially more dispersed, as reflected by greater intercellular distances (Figure 3G). Collectively, our findings demonstrate that the peritumoural region in malignant phyllodes tumours is not a passive bystander but an immunologically active and spatially remodelled niche.

### 2.4 Malignant Phyllodes Tumours Exhibit a Distinct Immunoactivated Peritumoural Phenotype

**FIGURE 4:**
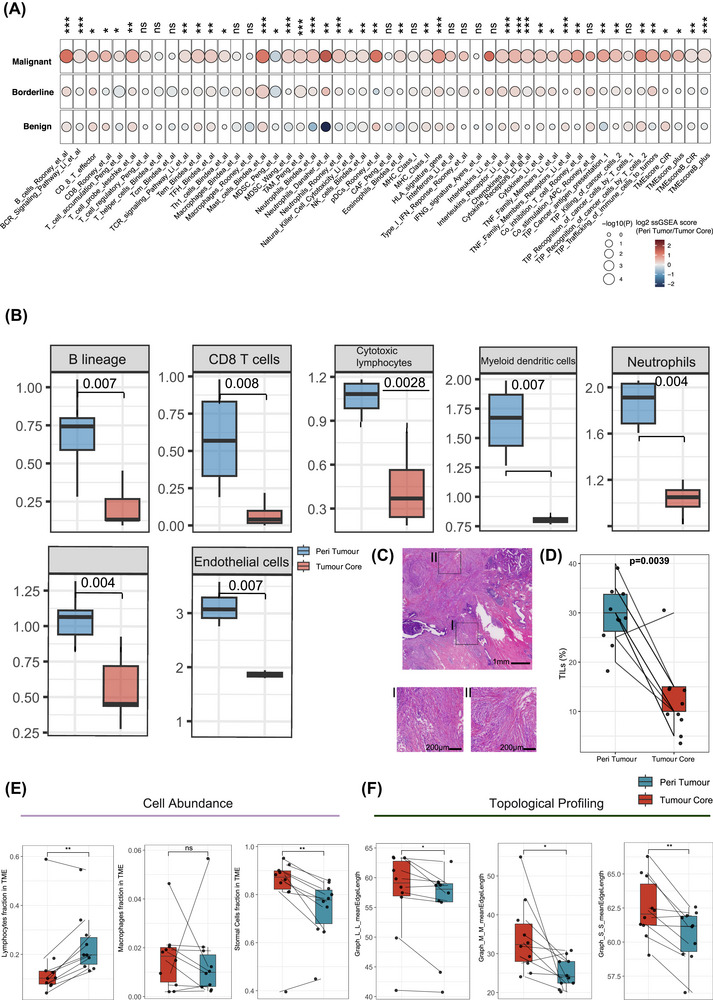
Malignant phyllodes tumours exhibit immune activated peritumoural niches and immune desert tumour cores. (A) Single sample gene set enrichment analysis (ssGSEA)‐based comparison of immune related signature scores between peritumoural tissues and tumour cores across benign, borderline, and malignant stages, showing marked enrichment of multiple immune signatures specifically in malignant peritumoural tissues. (B) MCP‐counter estimation of immune cell populations demonstrating differential cellular abundance between malignant peritumoural and core tissues. (C) Representative H&E images illustrating histopathological differences between malignant peritumoural and core regions. (D) Quantification of tumour infiltrating lymphocytes (TILs) showing significantly higher TIL proportions in malignant peritumoural tissues compared with their paired tumour cores. (E) Digital pathology‐based assessment of cell abundance. (F) Topological analysis comparing the spatial organization of immune and stromal cells between peritumoural and core regions.

To gain deeper insights into the biological features of malignant peritumoural tissues, we systematically compared the peritumoural regions and tumour cores of malignant phyllodes tumours from multiple analytical perspectives. First, a comprehensive set of immune response related signatures was collected and compared their enrichment between peritumoural and core tissues across benign, borderline, and malignant stages. Remarkably, significant differences were observed only in the malignant peritumoural tissues, where numerous immune related signatures were highly enriched compared with the corresponding tumour cores (Figure 4A), indicating that this immunoactive state is specific to the malignant peritumoural microenvironment. Beyond the overall immune signatures, four independent algorithms were applied to estimate immune cell abundance in malignant peritumoural versus core tissues. Consistent with the pathway analyses, multiple immune cell populations including T cells, B cells and dendritic cells, were significantly enriched in the peritumoural compartments (Figure 4B, Figure S3A). To experimentally validate these computational findings, we quantified tumour infiltrating lymphocytes (TILs) on histopathological slides obtained from paired peritumoural and core regions. The proportion of TILs was significantly higher in malignant peritumoural tissues than in matched tumour cores (Figure 4C,D). This phenomenon was not observed in the benign (Figure S3B,C) or borderline (Figure S3D,E) stages, confirming that the immune activation is specific to malignant disease.

At the spatial level, digital pathology‐based analyses further characterized differences in cellular abundance and topological architecture between malignant peritumoural and core regions. In agreement with TIL quantification, malignant peritumoural areas showed a marked enrichment of lymphocytes (Figure 4E). Unexpectedly, topological mapping revealed a higher cellular clustering degree among lymphocytes, macrophages and stromal cells near the malignant tumour boundary compared with the tumour core (Figure 4F), suggesting complex immune‐stromal interactions at the invasive front. Taken together, multi‐layer analyses demonstrate that the peritumoural tissues of malignant phyllodes tumours exhibit a unique immunoactivated signature and distinctive spatial topology that sharply contrast with the corresponding tumour cores. Taken together, these results reveal a distinctive immune dichotomy in malignant phyllodes tumours, characterized by an immune enriched peritumoural region encasing an immune excluded tumour core, which maybe a spatial hallmark of malignant progression driven by intricate tumour‐stroma interactions.

### 2.5 Angiogenesis and Extracellular Matrix Remodelling Underlie the Immune Activation Phenotype in Malignant Peritumoural Tissues

**FIGURE 5:**
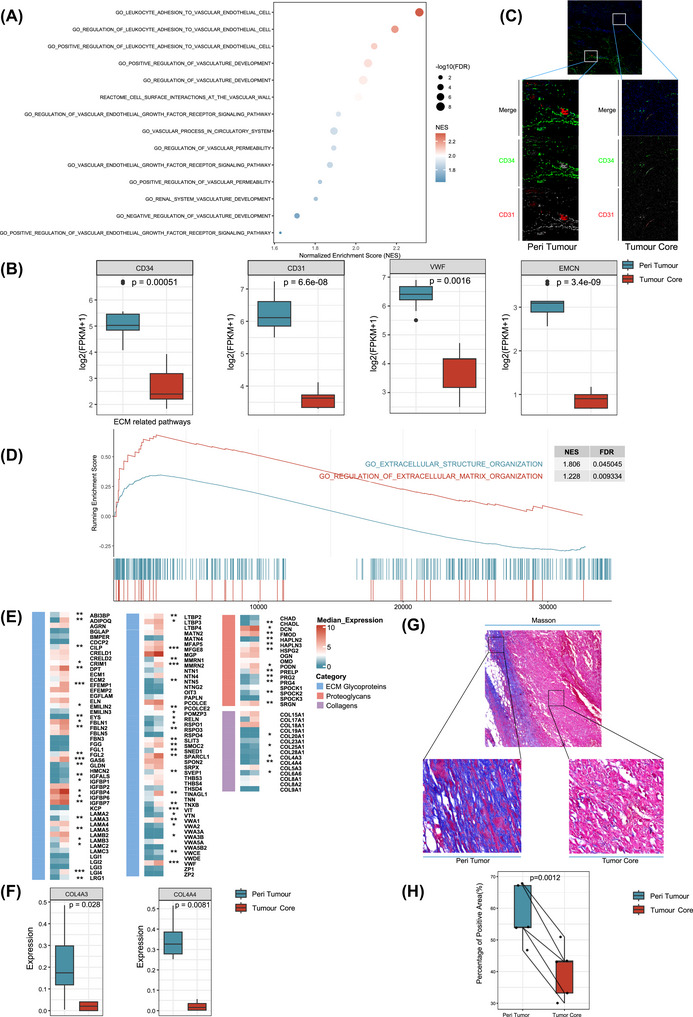
Enrichment of angiogenesis and extracellular matrix (ECM) remodelling in malignant peritumoural tissues. (A) Gene Ontology (GO) enrichment analysis showing significant upregulation of angiogenesis‐related pathways in malignant peritumoural tissues. (B) Comparative expression levels of vascular endothelial markers (CD31, CD34, and VWF) between peritumoural and core regions. (C) Multiplex immunofluorescence validating the enhanced protein expression and spatial distribution of CD31 and CD34 in malignant peritumoural tissues relative to tumour cores. (D) Gene set enrichment analysis (GSEA) identifying ECM remodelling pathways upregulated in malignant peritumoural regions. (E) Expression patterns of key genes involved in ECM‐related biological processes. (F) Upregulation of type IV collagen‐associated genes in malignant peritumoural tissues. (G) Representative Masson's trichrome stained images showing increased collagen deposition in malignant peritumoural compared with core tissues. (H) Quantification of collagen staining intensity confirming higher collagen content in peritumoural regions.

While malignant phyllodes tumours display pronounced immune activation in the peritumoural areas, their tumour cores remain largely immune excluded, suggesting that physical or molecular barriers may restrict immune cell infiltration. We therefore sought to elucidate the biological mechanisms that may contribute to this spatially compartmentalized immune pattern. GO enrichment analysis revealed significant upregulation of pathways related to angiogenesis and endothelial growth factor signalling in malignant peritumoural tissues (Figure 5A). Consistently, key vascular endothelial markers including *CD31*, *CD34* and *VWF*, were expressed at higher levels in peritumoural tissues compared with corresponding tumour cores (Figure 5B). These findings were further assessed by multiplex immunofluorescence, suggesting the enhanced vascularization in malignant peritumoural areas (Figure 5C).

In addition to angiogenic activation, GSEA analysis highlighted a prominent enrichment of extracellular matrix (ECM) remodelling pathways in the malignant peritumoural tissues (Figure 5D). ECM remodelling involves coordinated processes of collagen synthesis, proteoglycan turnover, and glycoprotein dynamics.^28^ Systematic profiling of key ECM related genes revealed significant upregulation of multiple components in malignant peritumoural tissues (Figure 5E), with particularly strong induction of type IV collagen associated genes (Figure 5F). Given the established biological and pharmacological importance of collagen in tumour microenvironment regulation, we further validated these findings experimentally. Masson staining of paired samples demonstrated markedly increased collagen deposition in malignant peritumoural tissues relative to tumour cores (Figure 5G,H). Collectively, these results suggest that enhanced angiogenesis and extensive ECM remodelling may key determinants of the ‘immune exclusion’ pattern in malignant phyllodes tumours.

### 2.6 Peritumoural Lymphocyte Distribution is Associated with Recurrence in Malignant Phyllodes Tumours

**FIGURE 6:**
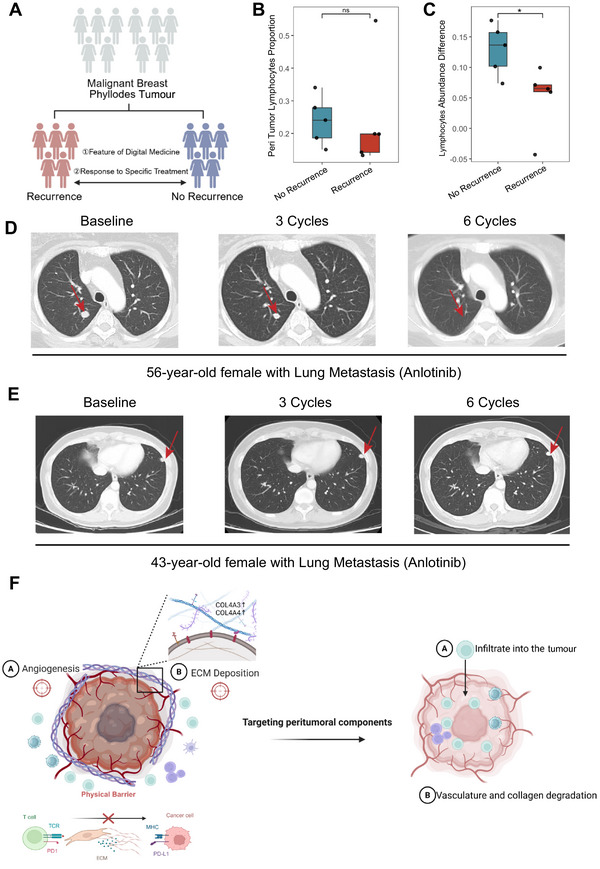
Translational implications of peritumoural immune features in malignant phyllodes tumours. (A) Study design for the prognosis related analysis. (B) Comparison of peritumoural lymphocyte abundance between non‐recurrent and recurrent patients. (C) Comparison of the peritumoural‐core lymphocyte difference between non‐recurrent and recurrent patients. (D‐E) Representative CT images from two relapsed patients treated with anlotinib, illustrating treatment response in patients with different peritumoural‐core lymphocyte differences. Arrows indicate target lesions. (F) Integrated summary of the molecular and microenvironmental features of malignant phyllodes tumours and potential therapeutic targets.

To investigate whether peritumoural immune features are associated with prognosis and treatment response, we analysed malignant phyllodes tumour patients with different clinical courses. Five patients who developed postoperative recurrence and five patients without recurrence were evaluated to quantify lymphocyte abundance of tumour core and peritumoural regions (Figure 6A). Absolute peritumoural lymphocyte abundance did not differ significantly between recurrent and non‐recurrent patients (Figure 6B). However, when focusing on the difference in lymphocyte abundance between peritumoural and tumour core regions (peritumoural minus tumour core), we found that patients with a larger peritumoural–core lymphocyte difference had a lower likelihood of recurrence (Figure 6C). A possible interpretation is that a stronger peritumoural lymphocyte enrichment relative to the tumour core may reflect a more effective immune containment phenotype at the tumour boundary. Thus, the gradient captures spatial organization rather than bulk immune quantity alone.

We further explored whether the peritumoural–core lymphocyte difference might relate to therapeutic response in the relapsed setting. Two recurrent patients who received the anti‐angiogenic agent anlotinib were examined with longitudinal CT imaging. Patient 1, who exhibited the largest peritumoural–core lymphocyte difference, demonstrated marked shrinkage of the target lesion after 3 and 6 cycles of anlotinib (Figure 6D). In contrast, Patient 5, whose peritumoural and tumour‐core lymphocyte abundances were similar, showed limited radiographic regression of pulmonary metastatic lesions after 6 cycles of anlotinib (Figure 6E).

Although only a few treated cases were available, these findings suggest that a larger peritumoural–core lymphocyte gradient may reflect stronger immune compartmentalization at the tumour boundary. This feature may be associated with improved response to anti‐angiogenic therapy after relapse.

## 3 DISCUSSION

Numerous studies have sought to elucidate the malignant progression of breast phyllodes tumours from various biological perspectives. The series of studies by Nie et al. revealed that interactions between macrophages and phyllodes tumour stromal cells play a pivotal role in driving malignant transformation, identifying key molecules such as *miR‐21^9^*, *CCL18^10^* and *CCL5^17^*. Another study established a patient‐derived xenograft (PDX) model to screen novel agents targeting critical signalling pathways involved in the progression of malignant phyllodes tumors.^29^ In addition, Li et al. explored the spatial heterogeneity within malignant phyllodes tumours and demonstrated that the interaction between *COL4A1/2* and *ITGA1/B1* is a key driver of malignant evolution.^30^ Despite these advances, the peritumoural region‐a structural and biological compartment known to profoundly influence tumour invasion and microenvironmental remodeling,^31^, ^32^ has not been systematically investigated in breast phyllodes tumours.

In this study, we established a comprehensive spatial and molecular landscape of phyllodes tumour progression by integrating multi‐regional transcriptomic and histopathological analyses across different stages. Our data reveal profound spatial heterogeneity between the tumour core and its surrounding tissues. Notably, malignant phyllodes tumours displayed a distinct immune profile, characterized by an immune activated peritumoural niche encasing an immune excluded tumour core. This spatial contrast underscores the emergence of local immune activation at the invasive front concurrent with immune exclusion within the tumour mass. We further identified features of angiogenesis and extracellular matrix remodelling associated with this immune barrier, suggesting that structural and vascular changes in the malignant microenvironment may impede effective immune infiltration (Figure 6F).

Our study revealed a distinct pattern of immune exclusion in malignant phyllodes tumours. Previous reports have described malignant phyllodes tumours as immune excluded, consistent with the findings of Nozad et al., who detected no tumours with high tumour mutational burden (TMB) among a cohort of 24 MPT cases.^33^ In contrast, our results suggest that a substantial portion of immune cells may be spatially sequestered outside the tumour mass, forming a peritumoural “immune reservoir”. This immune confinement appears to be closely associated with enhanced angiogenesis and collagen deposition, which likely create structural and biochemical barriers to immune infiltration. Similar phenomena have been documented in other malignancies. In glioblastoma, regions with elevated angiogenesis activity show pronounced immune cell aggregation and hold potential therapeutic relevance following surgery.^34^ Likewise, in hepatocellular carcinoma, peritumoural collagen accumulation and ECM remodelling are recognized contributors to immune exclusion; targeting collagen cross‐linking can enhance T cell infiltration and improve treatment outcomes.^35^ Importantly, the vascular abnormalities and collagen barriers observed in malignant phyllodes tumours likely function in concert rather than in isolation. Aberrant angiogenesis may trigger hypoxia and the subsequent activation of TGF‐β signalling, which promotes the differentiation of cancer associated fibroblasts and stimulates further collagen synthesis and cross‐linking.^36^ Conversely, excessive collagen deposition and matrix stiffening can compress and destabilize nearby vessels, exacerbating local hypoxia and vascular dysfunction.^37^ This reciprocal feedback between the vasculature and ECM may constitute a central mechanism maintaining the immune‐excluded microenvironment in malignant phyllodes tumours.

The observed immune exclusion phenotype in malignant phyllodes tumours may hold substantial translational and therapeutic relevance. In our study, patients with a more pronounced spatial immune segregation phenotype may have a lower risk of recurrence and may derive greater benefit from anti‐angiogenic therapy if relapse occurs. Preclinical evidence from other cancer models has demonstrated that targeting extracellular matrix remodelling and vascular abnormalities can effectively convert immune excluded tumours into immune responsive ones. For instance, Wan et al. showed that inhibiting *IGF1R* mediated collagen synthesis, in combination with immune checkpoint blockade, markedly enhanced antitumor immunity in triple negative breast cancer.^38^ Likewise, Khan et al. reported that blocking integrin α5β1 reduced fibronectin fibrillogenesis, improved endothelial permeability and facilitated CD8^+^ T cell transmigration, ultimately potentiating the efficacy of PD‐L1 blockade in the E0771 breast cancer model.^39^ Taken together with our findings and prior studies, larger future cohorts are warranted to investigate the relationship between peritumoural immune exclusion and the efficacy of anti‐angiogenic therapy. In parallel, the rationale for combining anti‐angiogenic agents with immune checkpoint inhibitors should be systematically evaluated.

This study has several limitations. First, while the sample size remains limited, multi‐regional sampling from three distinct tumour sites per patient was employed to compensate for this limitation and to better represent intratumoral heterogeneity. Second, although digital pathology allowed us to quantify immune and stromal cell abundance and spatial topology across distinct tumour regions, the use of high resolution spatial transcriptomics in future studies will provide a more refined understanding of cellular interactions between the tumour core and peritumoural compartments. Finally, the translational hypotheses proposed here require confirmation using functional perturbation of ECM remodelling and angiogenic pathways in relevant experimental systems, such as 3D patient‐derived models with anti‐angiogenic or anti‐fibrotic interventions.

Collectively, these findings present a spatial perspective on how malignant phyllodes tumours are associated with the surrounding microenvironment during progression. The unique characteristics of the peritumoural region may hold the key to breaking therapeutic resistance and opening new avenues for the treatment of malignant phyllodes tumours.

## 4 METHODS

### 4.1 Patients and Tissue Samples

This study was approved by the Institutional Review Board of Fudan University Shanghai Cancer Center (ethics number: 2301268‐17). All cases were recruited at Fudan University Shanghai Cancer Center from 2019 to 2022, were  > 18 years old, and had newly diagnosed, histologically confirmed primary breast phyllodes tumours. Biosamples were collected and stored as previously described.^40^ In this study, 22 cases of breast phyllodes patients with matched tumour and adjacent non‐neoplastic tissues meeting quality criteria were included. The non‐neoplastic tissues were obtained from lobectomy specimens, collected along the area extending from the tumour towards the resection margin.

### 4.2 Sample Processing for Genomic DNA and Total RNA Extraction

For quality control, fresh frozen tumour tissues were macrodissected. Genomic DNA was extracted from fresh frozen tissues and peripheral blood using the TGuide M24 system (Tiangen). DNA purity and concentration were assessed by measuring absorbance at 260 and 280 nm with a NanoDrop 2000 spectrophotometer (Thermo Scientific, Wilmington, DE, USA). Samples with A260/A280 ratios between 1.6 and 1.9 were considered suitable for downstream analyses. Total RNA was subsequently isolated from RNAlater‐preserved tissues using the miRNeasy Mini Kit (Qiagen, #217004) in accordance with the manufacturer's instructions. RNA integrity was evaluated on an Agilent 4200 Bioanalyzer with RNA ScreenTape (Agilent Inc.), and RNA concentrations were quantified using a NanoDrop ND‐8000 spectrophotometer (Thermo Fisher Scientific Inc.).

### 4.3 RNA‐Sequencing Data Processing

RNA‐sequencing (RNA‐seq) data were generated using a Ribo‐depletion protocol and processed through the NextSeq500 2×75 bp High Output platform, followed by quality assessment. Raw paired‐end FASTQ files were first trimmed and filtered with fastp. The cleaned reads were then aligned to the human reference genome (hg19) using HISAT2, and the resulting SAM files were converted to sorted BAM files with SAMtools. Gene‐level read counts were subsequently quantified against the hg19 GTF annotation from the University of California, Santa Cruz (UCSC) using HTSeq. The resulting expression matrix contained per‐sample gene identifiers and corresponding read counts. For downstream analyses, count data were normalized to the median total read count across samples and transformed to log_2_ scale.

### 4.4 WES Data Processing

Following alignment and quality assessment, somatic variants were identified by applying the Sentieon TNscope algorithm, using matched normal samples as controls. Detected variants were further annotated with ANNOVAR (version 2020.06) against multiple public databases including dbSNP, 1000 Genomes, gnomAD, and ClinVar to classify known and novel mutations.

### 4.5 Differential Gene Expression and Pathway Enrichment Analysis

Differentially expressed genes (DEGs) were defined as those exhibiting an absolute log_2_ fold change greater than 0.5 and an adjusted *p*‐value (Benjamini–Hochberg correction) below 0.05. Pathway enrichment analysis was performed using the R package GSVA,^41^ which computed pathway enrichment scores for each sample. Canonical pathways were derived from the Molecular Signatures Database (MSigDB), including REACTOME, GO, KEGG, and HALLMARK pathway sets.

### 4.6 Immune Cell Infiltration Inference from Gene Expression Profiles

The relative abundance of immune cell populations within the tumour microenvironment was inferred from the RNA‐seq expression data using MCP‐Counter, EPIC, quanTIseq, and xCell deconvolution algorithm.^42^, ^43^, ^44^, ^45^ All analyses were performed with default parameters as recommended by the developers.

### 4.7 Evaluation of TILs

Discrepancies between the two observers were resolved by joint review using a multiheaded microscope to reach a consensus score. The percentage of stromal TILs was assessed within the borders of the invasive tumour area, excluding regions of necrosis, fibrosis, or normal breast tissue. TIL density was quantified as the proportion of mononuclear inflammatory cells occupying the stromal compartment adjacent to tumour cells.^46^

### 4.8 Digital pathology

To identify cell types within the tumour microenvironment based on whole‐slide images (WSIs), we applied HoVer‐Net, a deep learning model designed for simultaneous nuclear segmentation and classification on H&E‐stained slides.^47^ The network adopts a dual‐branch architecture, in which one branch performs nuclear instance segmentation through a fully convolutional network (FCN) to delineate precise nuclear boundaries, while the other branch utilizes a convolutional neural network (CNN) to assign categorical labels to the segmented nuclei. This framework enables accurate recognition of multiple nuclear phenotypes, including immune and stromal cells. HoVer‐Net was pre‐trained on a large set of annotated H&E images using a multitask loss function that jointly optimizes segmentation and classification accuracy. In the present study, we focused primarily on immune and stromal cell populations, as these represent the key functional components of the tumour microenvironment. The detected nuclei corresponding to these cell types were extracted and subsequently subjected to topological analysis to characterize their spatial distribution and interactions. To ensure the reliability and biological relevance of tumour microenvironment (TME) features derived from the WSI‐based analysis, the identified cellular classifications were independently reviewed and validated by experienced pathologists.

### 4.9 Identification of Topological Features

To quantify intercellular spatial organization at the single cell level, we employed the single cell morphological and topological profiling (sc‐MTOP) framework. This graph‐based analytical approach models spatial interactions between specific cell types by constructing pairwise graphs that represent potential cellular relationships. For each graph, edges were established using a k‐nearest neighbour algorithm under the assumption that cells in close spatial proximity are more likely to interact. Distances were computed in a multidimensional feature space, enabling efficient identification of neighbouring cells. To minimize noise and spurious connections, redundant edges were filtered using an empirically determined 25 µm threshold. This radius was selected in accordance with prior cell‐graph computational pathology studies,^48^, ^49^ where limiting edges to a small spatial neighbourhood is used to capture short range tissue organization and local microenvironmental context while avoiding long distance links that are less likely to represent biologically meaningful interactions in histology sections. After generating these pairwise graphs, we derived comprehensive topological descriptors for each cell type interaction. Extracted features included:

1. Edge‐length metrics—MinEdgeLength and MeanEdgeLength, describing the minimum and average distances between connected cells;
2. Nsubgraph, indicating the number of cells within the subgraph to which each cell belongs; and
3. Degree, reflecting the number of direct connections between a cell and its neighbouring interacting cell type. Additionally, higher‐order graph‐theoretic measures, such as the clustering coefficient and betweenness centrality, were calculated to capture global structural complexity and identify hub‐like cells within the network. All feature matrices were concatenated to generate holistic topological profiles of the tumour microenvironment. Further methodological details of the sc‐MTOP framework are provided in the referenced publication.^24^

### 4.10 Masson Staining

Masson's trichrome staining was conducted with a commercial trichrome staining kit following the manufacturer's guidelines. Quantitative analysis of the stained sections was carried out using PRISM software, and the proportion of positively stained regions was calculated to assess the extent of Masson staining.

### 4.11 Multiplex immunofluorescence

Multiplex immunofluorescence (mIF) was performed on FFPE human tumour tissue sections. Slides were deparaffinized, rehydrated and subjected to heat‐induced antigen retrieval (Tris‐EDTA, pH 9.0). Sections were then permeabilized and blocked with protein blocking buffer. mIF staining was conducted using the Think Color staining kit according to the manufacturer's protocol. Primary antibodies included anti‐CD31 (clone JC70A, 1:50) and anti‐CD34 (clone QBEnd/10, 1:100). After incubation with secondary antibodies included in the kit, nuclei were counterstained with DAPI and slides were mounted with antifade medium. Images were acquired using identical settings across samples.

### 4.12 Statistical analysis

Normality of the variables was assessed using the Shapiro–Wilk test. For data following a normal distribution, comparisons between two groups were performed using either paired or unpaired Student's *t*‐tests. When the data were not normally distributed, the Wilcoxon rank‐sum test was applied instead. For analyses involving more than two groups, one‐way ANOVA or Kruskal–Wallis tests were used where appropriate. All statistical tests were two‐tailed, with significance thresholds set at *p* < 0.05. The significance levels were indicated as follows: *p* < 0.05 (*), *p* < 0.01 (**), *p* < 0.001 (***), *p* < 0.0001 (****) and ns for non‐significant differences. False discovery rate (FDR) values reported in the text are corrected for multiple testing, while *p*‐values are not corrected for multiple testing. All analyses were performed using R software (version 4.4.2).

## AUTHOR CONTRIBUTIONS

Conceptualization: Z.M.S., A.Y.C. Methodology: T.Q.G., X.R.W. Data curation: L.W., Q.Z., F.L.Q., C.C., G.H.D. Formal analysis: T.Q.G., X.R.W. Visualization: T.Q.G. and C.C. Writing‐Original Draft: T.Q.G., A.Y.C. Project administration: T.Q.G., L.W., Q.Z., F.L.Q., C.C., G.H.D., A.Y.C. Supervision: Z.M.S., A.Y.C.

## ETHICS STATEMENT

This study was approved by the Ethics Committee of Fudan University Shanghai Cancer Center (approval No. 2301268‐17). All procedures involving human participants were conducted in accordance with the Declaration of Helsinki.

## CONFLICT OF INTEREST STATEMENT

The authors declare no conflicts of interest.

## CONSENT FOR PUBLICATION

The authors have nothing to report.

## Supporting information




**Supplementary Figure 1**. Transcriptomic and genomic features of tumour cores across benign, borderline and malignant phyllodes tumours (see also Figure 2). (A) Differentially upregulated genes in borderline tumours compared with benign tumours. (B) Enriched biological pathways corresponding to the genes upregulated in borderline versus benign tumours. (C) Comparison of genomic alterations among benign, borderline and malignant tumours, illustrating the progressive accumulation of driver mutations. (D) Differentially expressed genes between MED12‐mutant and MED12‐wildtype malignant phyllodes tumours, highlighting enrichment of interferon signalling related transcripts. (E) Whole exome sequencing analysis of peritumoural samples. (F) Comparison of the number of copy number amplifications across phyllodes tumour stages. (G) Heatmap of oncogene copy number amplifications across samples.

**Supplementary Figure 2**. Digital pathology features of tumour cores across benign, borderline and malignant phyllodes tumours (see also Figure 2). (A) Representative images comparing HoVer‐Net automated cell type detection with pathologist manual annotations. (B) Correlation plots showing the concordance between manual annotation by pathologists and automated classification by HoVer‐Net across 20 representative fields of view (FOVs). For each FOV, the quantified counts of lymphocytes, macrophages and stromal cells were compared between the two approaches. (C–F) Quantitative digital pathology analysis depicting changes in cellular composition and stromal abundance across different stages.

**Supplementary Figure 3**. Peritumoural immune infiltration patterns across different stages. (A) Differences in immune cell infiltration abundance between peritumoural tissue and tumour core in malignant phyllodes tumours. Immune cell infiltration was deconvoluted from transcriptomic data using three independent algorithms (EPIC, quanTIseq and xCell). (B‐C) Representative images and corresponding quantitative analysis of TIL density in benign phyllodes tumours comparing tumour cores with peritumoural regions. (D‐E) Representative images and quantitative comparison of TIL infiltration in borderline phyllodes tumours between tumour cores and adjacent peritumoural tissues.

## Data Availability

The datasets used and analysed during the current study are available in the Genome Sequence Archive (GSA) database under accession code PRJCA058780.
