# Supplementary figures and images for: Integrated multi‐regional multiomic profiling of breast phyllodes tumours reveals peritumoural immune activation and stromal remodelling
Source: Clin Transl Med. 2026 Mar 31;16(4):e70644. doi: 10.1002/ctm2.70644 (PMC13140471; doi:10.1002/ctm2.70644)

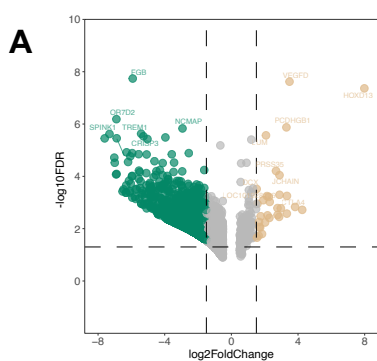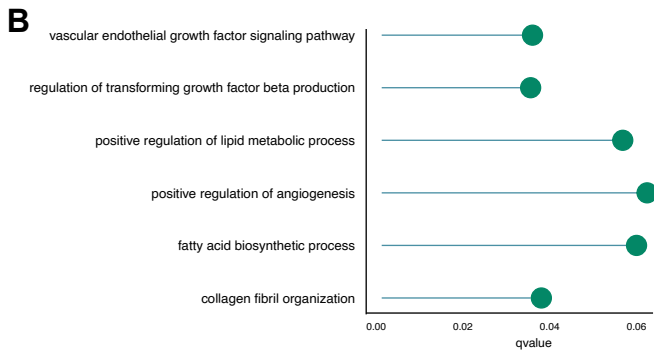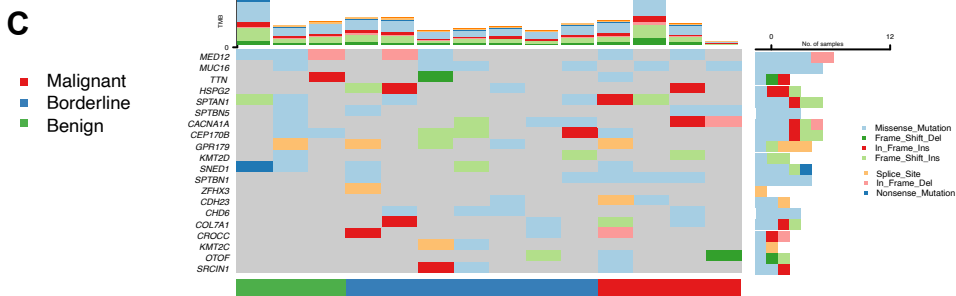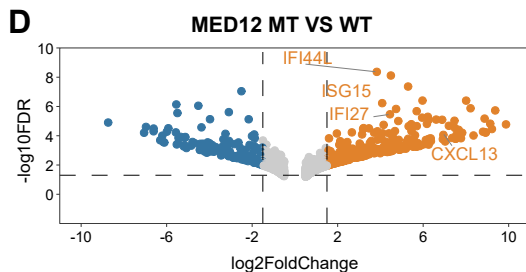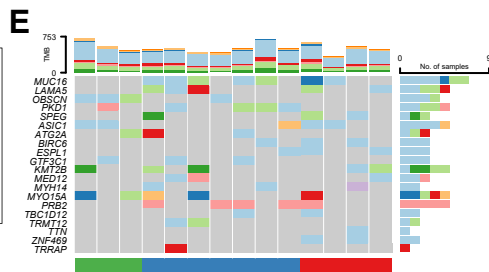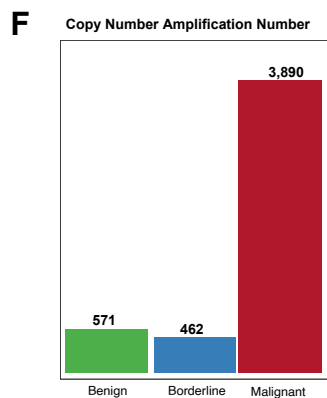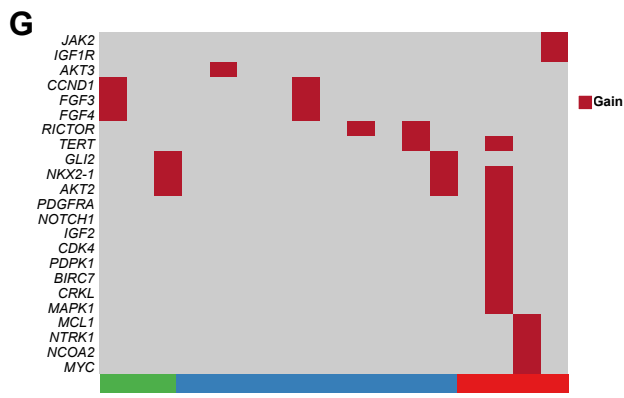

Supplement: Supplementary file 1 — Supplementary Figure 1. Transcriptomic and genomic features of tumour cores across benign, borderline and malignant phyllodes tumours (see also Figure 2). (A) Differentially upregulated genes in borderline tumours compared with benign tumours. (B) Enriched biological pathways corresponding to the genes upregulated in borderline versus benign tumours. (C) Comparison of genomic alterations among benign, borderline and malignant tumours, illustrating the progressive accumulation of driver mutations. (D) Differentially expressed genes between MED12‐mutant and MED12‐wildtype malignant phyllodes tumours, highlighting enrichment of interferon signalling related transcripts. (E) Whole exome sequencing analysis of peritumoural samples. (F) Comparison of the number of copy number amplifications across phyllodes tumour stages. (G) Heatmap of oncogene copy number amplifications across samples. [file CTM2-16-e70644-s002.pdf]

**Hover-Net**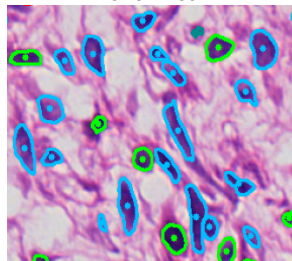**Pathologist**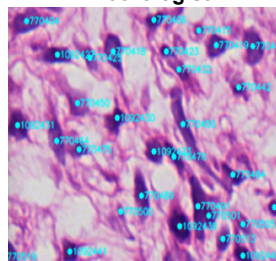**B**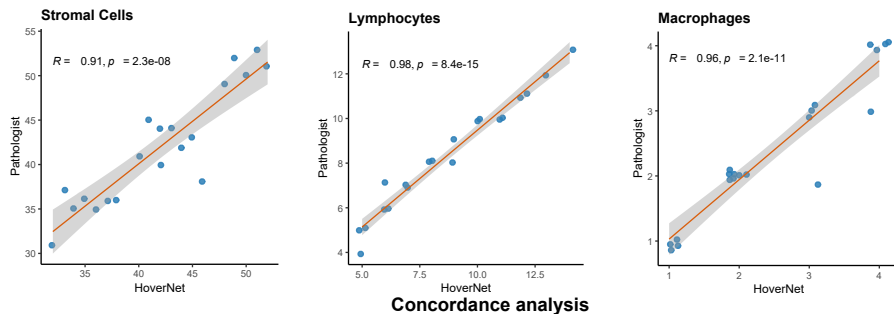**C**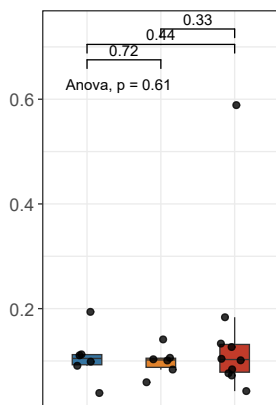**D**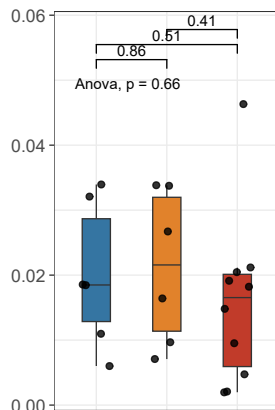

Benign

Borderline

Malignant

**E**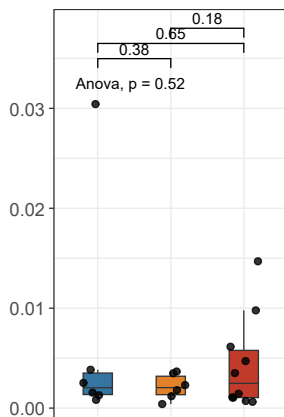**F**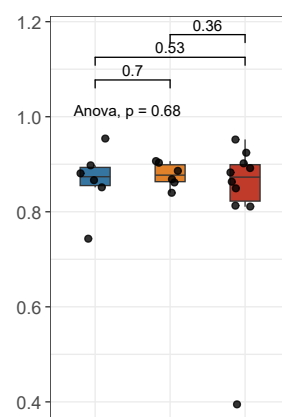

Supplement: Supplementary file 2 — Supplementary Figure 2. Digital pathology features of tumour cores across benign, borderline and malignant phyllodes tumours (see also Figure 2). (A) Representative images comparing HoVer‐Net automated cell type detection with pathologist manual annotations. (B) Correlation plots showing the concordance between manual annotation by pathologists and automated classification by HoVer‐Net across 20 representative fields of view (FOVs). For each FOV, the quantified counts of lymphocytes, macrophages and stromal cells were compared between the two approaches. (C–F) Quantitative digital pathology analysis depicting changes in cellular composition and stromal abundance across different stages. [file CTM2-16-e70644-s001.pdf]

**A**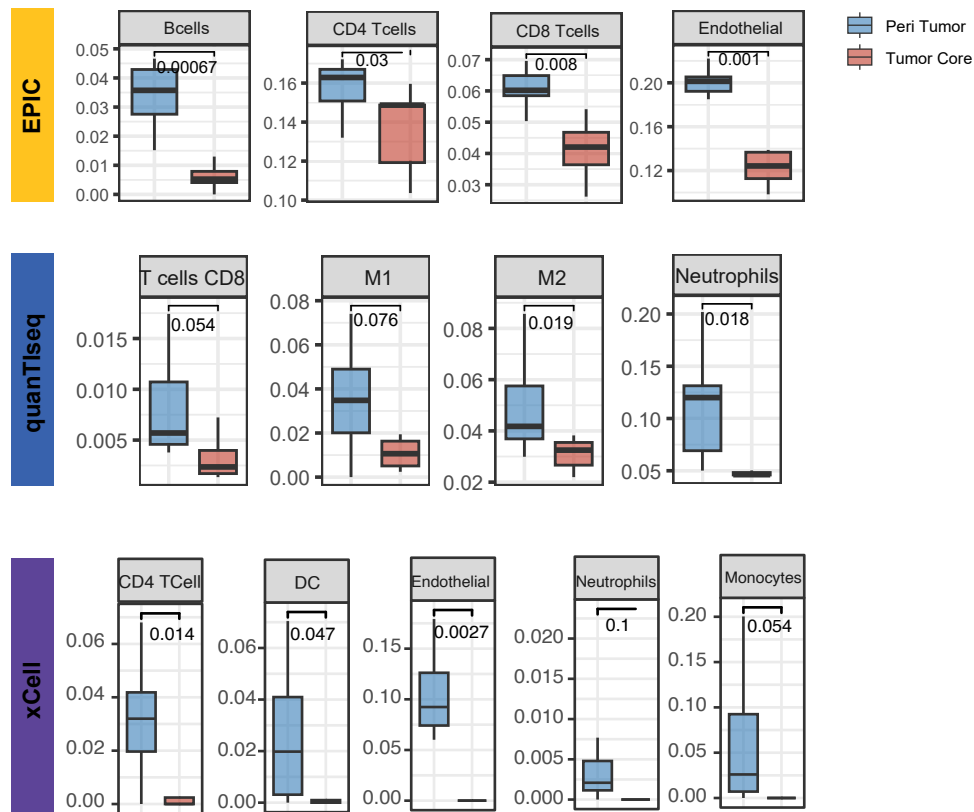**B**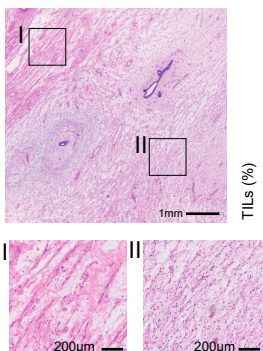**C**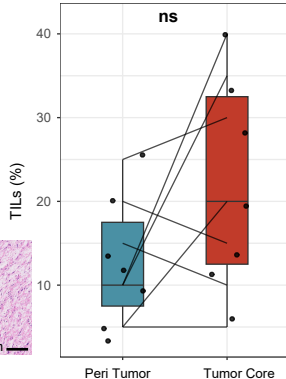**D**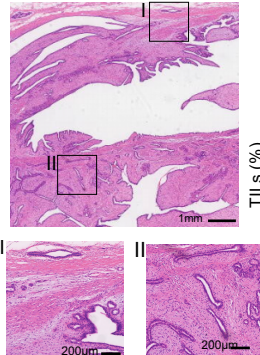**E**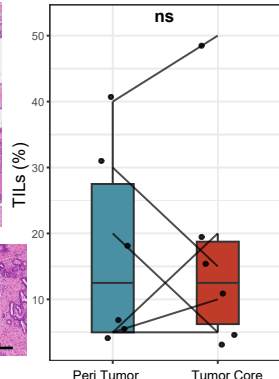

Supplement: Supplementary file 3 — Supplementary Figure 3. Peritumoural immune infiltration patterns across different stages. (A) Differences in immune cell infiltration abundance between peritumoural tissue and tumour core in malignant phyllodes tumours. Immune cell infiltration was deconvoluted from transcriptomic data using three independent algorithms (EPIC, quanTIseq and xCell). (B‐C) Representative images and corresponding quantitative analysis of TIL density in benign phyllodes tumours comparing tumour cores with peritumoural regions. (D‐E) Representative images and quantitative comparison of TIL infiltration in borderline phyllodes tumours between tumour cores and adjacent peritumoural tissues. [file CTM2-16-e70644-s003.pdf]
